# Hospitalized Women’s Perspective on Willingness-to-Screen for Cancers in Relation to Life Expectancy

**DOI:** 10.7759/cureus.25732

**Published:** 2022-06-07

**Authors:** Jocelyn Shubella, Gina Kauffman, Waseem Khaliq

**Affiliations:** 1 Division of Hospital Medicine, Johns Hopkins University School of Medicine, Baltimore, USA

**Keywords:** willingness-to-screen, hospitalized women, life-expectancy, patients’ preference, cancer screening

## Abstract

Objective: Life expectancy is an important tool for physicians and patients to determine when medical services for disease prevention should be rendered. Since patients’ preference is an important predictor for cancer screening compliance, incorporating life expectancy with cancer screening preferences becomes essential. The purpose of the study is to explore the mean life expectancy duration that hospitalized women expect in order to undergo cancer screening tests.

Methods: A cross-sectional bedside survey including the contingent valuation method was used to assess the mean life expectancy among 475 cancer-free hospitalized women aged 50-75 years, which justified their willingness to undergo cancer screening tests. The probit and logistic regression models were used for the analysis in October 2021.

Results: A total of 74% of women were willing to undergo cancer screening if the mean life expectancy was 24.3 months (SE = 12.8, p = 0.058). After adjustment for sociodemographic and clinical covariates, hospitalized women were willing to undergo cancer screening if the mean life expectancy was 26.6 months (SE = 13.3, p = 0.045). Race (African American and others vs Caucasians, OR = 2.34, 95% CI: 1.43-3.81) and annual household income <$20,000 (OR = 1.71, 95% CI: 1.02-2.86) were associated with the willingness to undergo cancer screening among hospitalized women.

Conclusion: The study’s findings suggest that hospitalized women value the prospect of cancer screening tests, given the mean life expectancy of approximately 27 months. Therefore, offering screening tests to nonadherent hospitalized women with a mean life expectancy of 2¼ years, especially to those at high risk for developing cancer, with low income, or women of color, may improve adherence to cancer screening recommendations.

## Introduction

Predicting a patient’s life expectancy is becoming an increasingly important tool for physicians and patients when determining which medical services should be rendered. This is of particular importance for cancer screening. It has been studied that patients with a life expectancy of less than five years are unlikely to derive any benefit from cancer screening [1]. Tools that predict long-term mortality and life expectancies, such as the Lee and Schonberg indices, have been used in shared decision-making models for choices regarding cancer screening and even cancer treatment in the geriatric population [2,3]. The Lee index is a four-year mortality index that was derived from community-dwelling adults over 50-years old who were interviewed in the Health and Retirement Survey in 1998. Similarly, the Schonberg index predicts five-year mortality in a heterogeneous sample of adults over the age of 65, who completed the National Health Interview Survey between 1997 and 2000 [3]. A combined Lee-Schonberg index has since been established, and these tools are readily available online at no cost to practitioners [4]. These indices have since been externally validated, extrapolated to accurately predict 10-year mortality, and are utilized in clinical practice to help guide decision-making for older adults [5-7].

Using these tools to help guide conversations with patients is of particular importance as many patients still cite their physicians’ recommendations and opinions as the most important factor for determining whether or not to pursue cancer screening [8]. This sentiment holds true not only for patients within the United States but also internationally [9]. Additionally, patients look to their physicians for guidance about the potential harms of screening with an emphasis that the patient has a choice in the decision of whether to pursue screening [10]. Patients even value it when inpatient physicians, with whom they may have a limited relationship, discuss breast and colorectal cancer screening with them while they are admitted to the hospital [11,12]. It is evident that open, honest communication between patients and physicians is what patients value most when it comes to decisions regarding the pursuit of cancer screening. When guiding a patient discussion regarding the utility of cancer screening, clinicians may find life expectancy tools useful, and understanding how these tools affect patients’ perspectives about screening remains to be studied. Our study aims to fill this gap in knowledge to determine if awareness of a patient’s life expectancy alters their perspectives on pursuing cancer screening. This study provides an insight into how hospitalized women value cancer screening in light of life expectancy. This study further seeks to establish the mean life expectancy duration acceptable to these hospitalized women for their willingness to undergo cancer screening tests.

## Materials and methods

Study design and sample

In this cross-sectional study design, 510 women aged 50-75 years who were cancer-free at baseline and admitted to the hospital under the care of the internal medicine service were enrolled at an academic medical center between December 1, 2014, and May 31, 2017. Detailed enrollment methods have been published [12]. Of these enrolled women, 475 (93%) believed that they should undergo routine screening tests that are designed to pick up cancer early and responded to a contingent valuation questionnaire designed to determine the mean life expectancy duration that these women considered acceptable for cancer screening tests. The contingent valuation methodology is used in health economics to estimate the value placed by patients for a healthcare service; inquiring about the life expectancy the patients would be willing to consider the service can provide useful information [13]. The willingness-to-screen (WTS) variable may approximate patient centeredness by allowing scientific evaluation to estimate how individuals value healthcare services [14].

Protocol and measures

The study coordinator used a bedside survey to collect data about the study populations’ sociodemographic information, medical comorbidities, cancer screening adherence, and risk for developing cancer. The study population believed that one should undergo routine cancer screening tests that are designed to pick up cancers early. We asked the following contingency question: “If it were known that a patient was to live for X months/years, do you think having undergone cancer screening tests like mammogram and screening colonoscopy is worthwhile and makes sense?” Each respondent was randomly assigned one of five possible durations for X (X = 6 months, 1 year, 3 years, 5 years, or 7 years). The respondents were instructed to answer either “Yes” or “No,” thereby indicating whether the stated life expectancy was acceptable. All study participants provided their informed consent for participation. The Institutional Review Board at Johns Hopkins School of Medicine (IRB00049608) approved the study ethics and protocol.

Statistical methods

Respondent characteristics are presented as proportions and means. Unpaired t-tests and Chi-square tests were used to compare women's WTS and not WTS toward cancer screening tests. T-test and Chi-square test determined the significance at a p-value ≤ 0.05. The probit regression model was used for the analysis of contingent valuation data to predict mean WTS [15]. A logistic regression model was used in the analysis to predict the sociodemographic and clinical variables that were thought to be associated with WTS. The data were analyzed in October 2021 using Stata, Version 13.1 (StataCorp LP., College Station, TX).

## Results

The mean age of the study population was 60.4 years (SD = 6.9), and 37% of study participants were African American. Characteristics of the study population, as stratified by WTS and not WTS, are shown in Table 1. There were no differences noted between the two groups except that women WTS were more likely to be African American as compared to not WTS women (Table 1).

**Table 1:** Characteristics of the study population ^Ŧ^ For some patients, the variables had missing values. ^*^ Chi-square, Fisher’s exact statistic (where at least 20% of frequencies were <5), and unpaired t-test statistic.

Characteristics^Ŧ^	Not Willing-to-Undergo Screening (N = 125)	Willing-to-Undergo Screening (N = 350)	p-value^*^
Age ≥ 60 years, n (%)	64 (51)	176 (50)	0.86
Race			0.004
Caucasians, n (%)	91 (73)	197 (56)	
African American, n (%)	31 (25)	143 (41)	
Others, n (%)	3 (2)	10 (3)	
Married or living with a partner, n (%)	86 (69)	240 (69)	0.96
High school or more years of education, n (%)	103 (82)	276 (79)	0.44
Employment status, n (%)			0.63
Employed	27 (22)	79 (23)	
Unemployed	9 (7)	23 (7)	
Retired	39 (31)	90 (26)	
Disability/unable to work	50 (40)	158 (45)	
Annual household income < $20,000, n (%)	44 (36)	163 (47)	0.06
Uninsured, n (%)	0 (0)	2 (1)	0.40
No primary care physician, n (%)	7 (6)	30 (9)	0.34
Presenting to hospital from home, n (%)	123 (98)	334 (98)	0.70
Admitted as observation, n (%)	10 (8)	16 (5)	0.17
Principle diagnosis by the system at admission, n (%)			0.30
General internal medicine	39 (31)	110 (31)	
Cardiovascular	15 (12)	59 (17)	
Pulmonary	23 (18)	63 (18)	
Gastrointestinal	21 (17)	39 (11)	
Neurology	1 (1)	6 (2)	
Nephrology	8 (7)	26 (7)	
Oncology	5 (4)	3 (1)	
Rheumatology	4 (3)	12 (3)	
Psychiatry	0 (0)	4 (1)	
Infectious disease	5 (4)	19 (5)	
Others	4 (3)	9 (3)	
Discharge from hospital to home, n (%)	123 (98)	338 (97)	1.00
Length of stay in days, mean (SD)	5 (3.8)	4.8 (5.5)	0.70
Alcohol use, n (%)	33 (26)	91 (26)	0.93

Table 2 describes variables pertaining to functional status, health behaviors and attitudes toward cancer screening, mortality risk as defined by the Lee and Schonberg scores, Charlson Comorbidity index (CCI), and medical comorbidity burden, stratified by WTS and not WTS. While a majority of variables were similar across the two groups, women in the WTS were more likely to have hypertension and coronary artery disease (p = 0.002 and 0.04, respectively).

**Table 2:** Health behavior for cancer screening, cancer risk stratification, and medical and functional disability burden of study population by WTS ^Ŧ^ For some patients, the variables had missing values. ^*^ Chi-square, Fisher’s exact statistic (where at least 20% of frequencies were <5), and unpaired t-test statistic. ^a^ Family history of a first-degree relative with colorectal cancer (fathers, mothers, brothers, sisters, daughters, and sons). ^b^ History of Lynch syndrome, familial adenomatous polyposis, or inflammatory bowel disease. ^c^ Family history of breast cancer was defined as breast cancer in first-degree relatives like mothers, sisters, or daughters. ^d^ Gail score was estimated using the National Cancer Institute Breast Cancer Risk Tool (http://www.cancer.gov/bcrisktool/). ^e^ Charlson comorbidity index (CCI) scores of 0, 1, 2, and 3 predicting 10-year survival rates of 93%, 73%, 52%, and 45%, respectively. ^f^ Comorbidities excluded diseases accounted for CCI and included hypertension, heart disease, hypercholesterolemia, atrial fibrillation, obstructive sleep apnea, depression, chronic hepatitis, hypothyroidism, nephrolithiasis, and anemia.

Characteristics^Ŧ^	Not Willing-to-Undergo Screening (N = 125)	Willing-to-Undergo Screening (N = 350)	p-value^*^
Ambulatory function			0.71
Ambulate without assistance, n (%)	80 (64)	208 (59)	
Ambulate with cane or walker, n (%)	40 (32)	127 (36)	
Chronic disability, wheelchair, or bedbound, n (%)	5 (4)	15 (4)	
Difficulty walking a quarter mile without help or equipment, n (%)	73 (58)	222 (63)	0.32
Difficulty with household chores, shopping, and getting around without help, n (%)	52 (42)	162 (46)	0.37
Difficulty managing finance due to health or memory issues, n (%)	8 (6)	44 (13)	0.07
Difficulty breathing or showering due to health or memory issues, n (%)	18 (14)	50 (14)	1.00
Difficulty pushing or pulling large objects due to health issues, n (%)	81 (65)	207 (59)	0.27
Currently on ≥5 prescription drugs, n (%)	79 (64)	239 (69)	0.31
Self-reported overnight hospitalization in the last 12 months, mean (SD)	2.5 (3.1)	2.4 (2.9)	0.71
Nonadherent to colorectal cancer screening, n (%)	35 (28)	86 (25)	0.45
First-degree relative with colon cancer,^a^ n (%)	9 (7)	44 (13)	0.14
High risk for colorectal cancer,^b^ n (%)	12 (10)	31 (9)	0.86
Nonadherent to breast cancer screening, n (%)	44 (36)	108 (31)	0.32
Family history of breast cancer​,​​​​​^c ^n (%)	19 (15)	64 (18)	0.49
5-year-risk prediction using Gail model ≥ 1.7%,^d^ n (%)	49 (39)	145 (41)	0.67
Current and ex-smoker, n (%)	70 (56)	224 (64)	0.11
BMI kg/m^2^, n (%)			0.09
Less than 25	26 (21)	76 (21)	
25-29.9	29 (23)	51 (15)	
≥30	70 (56)	223 (64)	
Age-adjusted CCI > 3,^e^ n (%)	48 (38)	142 (41)	0.67
Median Lee score (IQR)	5 (2-6)	5 (3-7)	
5-year mortality risk for median Lee score (%)	8	8	
10-year mortality risk for median Lee score (%)	23	23	
Life expectancy for median Lee score (years)	17.7-21.1	17.7-21.1	
Median Schonberg score (IQR)	6 (3-8)	6 (3-9)	
5-year mortality risk for median Schonberg score (%)	10	10	
10-year mortality risk for median Schonberg score (%)	26	26	
14-year mortality risk for median Schonberg score (%)	42	42	
Total comorbidities (excluding CCI), mean (SD)^f^	3 (1.9)	3.3 (1.8)	0.07
Coronary artery disease, n (%)	24 (19)	120 (34)	0.002
Hypertension, n (%)	88 (70)	278 (79)	0.04
Hyperlipidemia, n (%)	59 (47)	176 (50)	0.55
Anemia, n (%)	53 (42)	172 (49)	0.21
Obstructive sleep apnea, n (%)	10 (8)	47 (13)	0.15
Depression, n (%)	49 (39)	124 (35)	0.45
Hypothyroidism, n (%)	53 (16)	26 (15)	0.9
Atrial fibrillation, n (%)	14 (11)	35 (10)	0.73
Hypothyroidism, n (%)	21 (17)	52 (15)	0.67
Nephrolithiasis, n (%)	10 (8)	22 (6)	0.54

**Figure 1:**
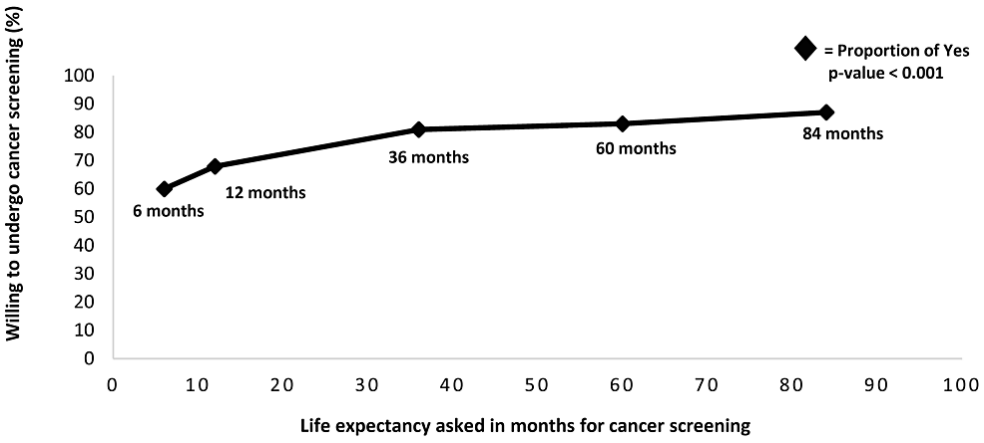
Proportion of hospitalized women willing to undergo cancer screening by life expectancy in months

For the study population, the median life expectancy for women to express a willingness to undergo cancer screening was 36 months or three years; 74% of women who were willing to undergo screening reported a mean life expectancy of 24.3 months (SE = 12.8, p = 0.058) determined using a probit regression model. After adjustment of 19 sociodemographic and clinical variables, the mean life expectancy to undergo screening tests was essentially unchanged at 26.6 months (SE = 13.3, p = 0.045). A direct relationship was noted between the proportion of women willing to consider screening tests and their reported life expectancy in months (Figure 1) in the contingent value question (p < 0.001).

We used logistic regression modeling to evaluate sociodemographic and clinical variables associated with WTS among hospitalized women. After adjustment of sociodemographic and clinical variables, the odds of willingness to undergo screening tests were 2.34 times higher for African American and other women (OR = 2.34, 95% CI: 1.43-3.81) as compared to Caucasian hospitalized women and 1.71 times higher among women with annual household income < $20,000 (OR = 1.71, 95% CI: 1.02-2.86) compared to women with annual household income > $20,000 (Table 3).

**Table 3:** Unadjusted and adjusted logistic regression analyses of variables associated with the willingness to undergo cancer screening tests among hospitalized women * Unadjusted model for individual risk factor prediction. ** Adjusted model for health-related medical issues thought to potentially influence the willingness to undergo cancer screening tests among hospitalized women age, race, marital status, education, employment status, annual household income, having no primary care provider, smoking status, ambulatory status, alcohol use, obesity, nonadherence to colorectal cancer screening, high risk for colorectal cancer, family history for colorectal cancer, nonadherence to breast cancer screening, high risk for developing breast cancer, family history for breast cancer, age-adjusted Charlson comorbidity index (CCI), and total comorbidities excluding CCI greater than 3.

Variables	Odds Ratio (95% CI)	
Predictor of willingness to undergo cancer screening tests	Unadjusted Model^*^	Adjusted Model^**^
Age < 60 years	1.04 (0.69–1.56)	0.89 (0.52–1.52)
African Americans and other races (versus Caucasians)	2.08 (1.33–3.25)	2.34 (1.43–3.81)
Married or living with a partner	1.01 (0.65–1.57)	1.70 (1.01–2.86)
Education less than high school	1.26 (0.74–2.13)	1.10 (0.61–1.97)
Unemployed/retired/disabled	0.95 (0.58–1.55)	0.80 (0.45–1.43)
Annual household income less than $20,000	1.54 (1.01–2.36)	1.71 (1.02–2.86)
No primary care provider	1.58 (0.68–3.70)	1.63 (0.65–4.10)
Current and ex-smoker (versus never smoker)	1.40 (0.92–2.12)	1.52 (0.96–2.41)
Ambulatory status (walk with cane or bedbound)	1.21 (0.80–1.85)	1.02 (0.62–1.67)
Alcohol use	0.98 (0.62–1.56)	0.95 (0.57–1.60)
Obesity (BMI ≥ 30 vs BMI < 30)	1.38 (0.91–2.09)	1.29 (0.82–2.02)
Nonadherence to colorectal cancer screening	0.84 (0.53–1.33)	0.88 (0.52–1.51)
High risk for colorectal cancer	0.92 (0.45–1.84)	0.81 (0.38–1.73)
Family history of colorectal cancer	1.83 (0.87–3.88)	2.03 (0.93–4.43)
Nonadherence to breast cancer screening	0.80 (0.52–1.24)	0.78 (0.48–1.28)
High risk for breast cancer	1.10 (0.72–1.67)	1.03 (0.58–1.84)
Family history of breast cancer	1.25 (0.71–2.18)	0.99 (0.48–2.01)
Age-adjusted CCI > 3	1.10 (0.72–1.66)	0.97 (0.57–1.66)
Total comorbidities > 3 (excluding CCI)	1.38 (0.90–2.11)	1.51 (0.91–2.52)

## Discussion

This study attempted to identify factors that affect women’s willingness to undergo cancer screening, particularly knowledge of their life expectancy as predicted by their Lee and Schonberg scores. We report hospitalized women who are cancer-free at baseline and are willing to undergo cancer screening tests, provided a mean life expectancy of approximately 27 months. We also found that women of color and women with low income were more willing to undergo cancer screening. We also found that longer life expectancies portended a greater willingness to undergo cancer screening as well.

Prior studies have shown that depression [16] and morbid obesity [17] in women are associated with poorer adherence to cancer screening guidelines. In hospitalized women, those with lower income status and a history of either smoking or stroke are less likely to comply with breast cancer screening [18]. In our study, many of these factors did not affect hospitalized patients’ willingness to undergo cancer screening, including those with a history of nonadherence to colorectal and breast cancer screening. However, our study showed that women with low income and women of color had a greater WTS compared to women of higher income and Caucasian women. Other studies have also found that the majority of hospitalized women, including those with lower incomes, would be willing to make a monetary contribution to complete screening mammography while hospitalized [19,20]. Hospitalizations provide a unique opportunity to target these historically nonadherent and low-income patient populations to receive inpatient cancer screening tests as they are not any less willing to undergo screening than their counterparts, and they even may be willing to pay for it [19]. Furthermore, studies have shown that screening tests can be safely done in the hospital setting [21-23].

The patients' functional status remains an important consideration for the decision of whether to pursue cancer screening as frailty indices have been shown to predict mortality as accurately as prognostic indices, such as Lee and Schonberg indices [24]. Our study did not find any difference in women’s WTS based on their functional statuses, such as their mobility and ability to perform activities of daily living (ADLs) and instrumental activities of daily living (iADLs); however, we did find that the longer a patient’s perceived life expectancy, the more willing they were to undergo cancer screening tests. It has been shown that patients with a higher functional status who have gastric, lung, or colorectal cancer are associated with having lower all-cause mortality from cancer and its treatments [25]. This is an important factor for providers who offer counseling regarding cancer screening as patients with higher functional status may need to be more directly targeted and convinced to undergo cancer screening. It is critical to capture this population while they still have an adequate functional status for both the prevention and treatment of cancer. It also emphasizes the need for providers to have candid conversations with their patients regarding life expectancy as this knowledge affects women’s willingness to undergo screening. A recently published study by Schoenborn et al. showed that there still clearly is an important role for screening mammography to reduce 10-year all-cause mortality, even when adjusted for various health comorbidities and functional status [26].

It is cited in the literature that women with poor access to health care are disproportionately less educated, belong to racial and ethnic minorities, or are from a low-income group [27]. Often the women from racial and ethnic minorities are diagnosed with breast cancer at more advanced stages and have higher mortality from breast cancer compared to Caucasian women, citing various reasons for this discrepancy including cost and lack of insurance, less access to care, poorer health literacy, language barriers, and cultural beliefs [28-29]. For instance, Austin et al. found that Hispanic women feel less susceptible to cancer and often do not undergo screening as a result [30]. Interestingly, our study demonstrated that women of color are actually more likely to express a willingness to undergo cancer screening during their hospitalization. As a result, hospitalizations provide a critical opportunity to capture these women who are willing to screen and otherwise may not be able to pursue cancer screening in the outpatient setting.

It has been studied that those who are socioeconomically disadvantaged and those of African American race have shorter and more variable lifespans. There are several reasons aside from genetics that have been postulated to account for these differences, including feelings of shame and indignity in their social status, which leads to stress and stress-related poor health behaviors like smoking. With more variable lifespans, African Americans have greater uncertainty about their future and life expectancy and are more likely to discount their future as a result. Therefore, if people of color or those from low-income groups perceive their life expectancy to be lower, this may negatively affect their willingness to undergo cancer screening tests. This has important implications when women of color and those of low income are more willing to undergo screening tests in our study; ultimately, if they perceive their life expectancy to be lower than their Caucasian or affluent counterparts, there may be a limit to their willingness to undergo screening. Additional studies should be performed to evaluate this concept further.

Several limitations of this study should be considered. First, this study was performed at a single institution in an urban setting, which may limit the generalizability of the results. Second, the survey was collected while patients were admitted to the hospital. Many factors affect how patients are feeling on any given day during their hospitalization, which may have altered their responses, possibly subjecting them to observation bias. However, in our experience caring for hospitalized patients for many years at multiple hospitals, patients are generally receptive and willing to undergo tests recommended by the physicians caring for them in the hospital [11,21]. Third, the study used hypothetical questions and the reliability of response can only be evaluated in real-time situations. However, studies have shown that hospitalized women not only value the opportunity to be screened for cancers, but when offered, they will undergo inpatient screening [19,21]. Fourth, our study included only women. However, this can also be counted as a strength of the study considering that women perceive cancer risk differently as compared to men and are more likely to be nonadherent to certain cancer screening, such as lung and colorectal cancers. Lastly, as the data was collected at one point in time, it is unknown if the patients’ WTS remains consistent over time.

## Conclusions

Hospitalizations are events where patients reflect upon their health and have more frequent encounters with healthcare providers. Many studies have corroborated that hospitalizations may be the ideal juncture to screen for certain types of cancers such as breast, colorectal, and cervical cancers as many barriers exist for patients to perform cancer screening in the outpatient setting. Women of color and those with low income are two populations of patients who are more willing to undergo cancer screening tests in the hospitalized setting. It is critical to utilize hospitalizations as an opportunity to capture these patients for cancer screening as these populations desire to receive cancer screening tests but historically face more barriers to pursing screening in the outpatient setting compared to respective counterparts.
